# Data‐Driven Investigation of Traditional Chinese Medicine Approaches for Optimizing Multiple Clinical Parameters in Esophageal Cancer Treatment

**DOI:** 10.1155/ijog/6667025

**Published:** 2025-12-15

**Authors:** Ying Fang, Jifang Peng, Dandan Yang, Hairong Liu, Tianle Chen, Juan Cao

**Affiliations:** ^1^ Department of Thoracic Surgery, The First Affiliated Hospital With Nanjing Medical University, Nanjing, China, njmu.edu.cn

**Keywords:** blood glucose fluctuation, comfort level, esophageal cancer, nursing service quality, psychological stress, radical surgery, traditional Chinese medicine appropriate technology

## Abstract

**Background:**

The purpose of the study is to evaluate the comparative effects of conventional perioperative care and conventional care plus Traditional Chinese Medicine (TCM) appropriate technology on blood glucose fluctuation, comfort level, and stress response in postoperative esophageal cancer patients.

**Methods:**

This study included 100 patients who underwent radical esophagectomy in our hospital’s thoracic surgery department from January 2023 to June 2024. Patients were randomly divided into a study group and a control group, with 50 cases in each group, using a computer‐generated random number table (generated via SPSS 22.0 software) to ensure randomization integrity. The control group received conventional perioperative care, while the study group received TCM appropriate technology in addition to conventional care. Blood glucose fluctuations were monitored 1 day before surgery, and on postoperative Days 1, 3, 7, and 14. On the day before surgery and postoperative Day 14, the General Comfort Questionnaire (GCQ) and Perceived Nursing Service Quality (PNSQ) scale were used to evaluate patients’ comfort levels and perception of service quality. The Connor–Davidson Resilience Scale (CD‐RISC) was used to assess psychological stress state. Physiological stress was evaluated by analyzing adrenocorticotropic hormone (ACTH) and cortisol (Cor) levels.

**Results:**

Significant differences in blood glucose fluctuation values were observed between the study group and control group (between‐group F = 9.457, *p* < 0.001). Significant differences also existed across different time points (time point F = 6.542, *p* < 0.001), with a significant interaction effect between group and time point (group∗time point F = 10.055, *p* < 0.001). Although blood glucose fluctuation levels in both groups showed a downward trend, the study group demonstrated significantly lower blood glucose fluctuation values than the control group at all time points: 1 day before surgery, and on postoperative days 1, 3, 7, and 14 (*p* < 0.05). The study group’s total GCQ score on postoperative Day 14 (89.36 ± 12.40) was higher than the control group (73.07 ± 10.14), with all intergroup differences in scores across the four dimensions being statistically significant (*p* < 0.001). The study group’s CD‐RISC score on postoperative Day 14 (74.23 ± 10.3) was higher than the control group (65.34 ± 9.06), while ACTH (12.23 ± 1.68 ng/L) and Cor (9.46 ± 1.29 ng/L) levels were lower than the control group (14.41 ± 1.98 and 10.22 ± 1.41 ng/L, respectively). All differences were statistically significant (*p* < 0.001).

**Conclusion:**

TCM appropriate technology significantly reduces blood glucose fluctuations in patients undergoing radical esophagectomy, improves patient comfort levels, and positively impacts psychological stress (via higher Connor–Davidson Resilience Scale [CD‐RISC] scores: 74.23 ± 10.3 vs. 65.34 ± 9.06) and physiological stress (via lower adrenocorticotropic hormone [ACTH]: 12.23 ± 1.68 vs. 14.41 ± 1.98 ng/L; cortisol [Cor]: 9.46 ± 1.29 vs. 10.22 ± 1.41 ng/L).

## 1. Introduction

According to data released by the International Agency for Research on Cancer of the World Health Organization in 2022, the incidence of esophageal cancer was approximately 510,000 cases, with a mortality rate of about 445,000 cases [1], severely affecting people’s health and life safety [2]. Surgery remains the primary treatment modality for esophageal cancer and is considered the main curative approach in clinical practice [3, 4]. The early postoperative period requires fasting for 7 days, and the significant surgical trauma leads to reduced digestive function. Simultaneously, surgical stress enhances catabolic metabolism in patients, resulting in malnutrition [5]. Therefore, postoperative nutritional support should be provided to patients with esophageal cancer to improve metabolic consumption and reduce stress response. However, the high‐protein, high‐calorie nutritional solutions typically chosen for nutritional support may cause difficulties in blood glucose control [6].

Unstable blood glucose levels can produce multiple adverse effects on postoperative recovery. First, blood glucose fluctuations impair immune function, significantly increasing infection risk. Second, this metabolic dysregulation may further induce various postoperative complications, such as delayed wound healing or cardiovascular events. Additionally, patients’ comfort during hospitalization may be reduced because of frequent blood glucose monitoring and interventional measures. From a clinical outcome perspective, poor glycemic control often leads to prolonged hospitalization and may even increase the risk of in‐hospital mortality [7, 8]. Current glucose management for patients with esophageal cancer predominantly focuses on insulin pharmacotherapy [5], but medication‐based approaches carry hypoglycemic risks and have limitations in regulating metabolic disorders [9]. Notably, insulin pharmacotherapy also fails to address stress‐induced metabolic disturbances, which are common in patients with postoperative esophageal cancer due to surgical trauma.

Traditional Chinese Medicine (TCM), as an essential component of China’s traditional medical system, possesses unique advantages and plays a significant role in blood glucose management [10]. TCM appropriate technology, which falls under external treatment methods, includes commonly used nursing techniques such as Chinese herbal external applications, auricular seed pressure therapy, acupoint massage, and acupoint stimulation [11]. Auricular seed pressure and acupoint application were chosen for their reported roles in glycemic regulation and stress relief in perioperative settings, as they can act on key acupoints related to metabolism and neuroendocrine function. These techniques can improve insulin resistance and stabilize blood glucose fluctuations through holistic regulation, while also featuring simplicity, efficacy, convenience, and cost‐effectiveness [12], providing new perspectives for perioperative glycemic management. Therefore, this study aims to investigate the effects of TCM appropriate technology on blood glucose fluctuations, comfort levels, and stress responses in patients undergoing radical esophagectomy. We hope to validate the effectiveness of TCM appropriate technology in managing blood glucose levels in patients with esophageal cancer, provide scientific evidence for clinical practice, and offer more comprehensive, personalized medical services to patients.

## 2. Method

### 2.1. General Data

This study included patients who underwent radical esophagectomy in our hospital’s thoracic surgery department from January 2023 to June 2024. All included patients met the following criteria: (1) pathologically confirmed diagnosis of esophageal cancer; (2) scheduled for esophageal cancer surgery; (3) aged 18–75 years; (4) absence of serious organic diseases of the heart, lungs, liver, or kidneys; (5) normal cognitive function; (6) informed consent to participate in the study voluntarily. Exclusion criteria were as follows: presence of mental disorders or cognitive dysfunction.

A total of 100 patients were enrolled and randomized into a study group and a control group using a random number table, with 50 patients in each group. Study group (*n* = 50): 30 males and 20 females; mean age 56.2 ± 8.7 years; disease course 6.4 ± 3.1 months; TNM staging: stage I (eight cases), stage II (18 cases), stage III (24 cases); body mass index 18.8 ± 2.6 kg/m^2^. Control group (*n* = 50): 28 males and 22 females; mean age 58.1 ± 9.3 years; disease course 7.1 ± 2.8 months; TNM staging: stage I (six cases), stage II (21 cases), stage III (23 cases); body mass index 18.2 ± 3.0 kg/m^2^. Comparison of general data between the two groups showed no statistically significant differences (*p* > 0.05).

### 2.2. Methods of Perioperative Blood Glucose Management

Both groups received conventional measures for blood glucose control, including blood glucose monitoring and hypoglycemic medications. The study group received TCM appropriate technology in addition to conventional measures.

Conventional measures: (1) Initial screening upon admission included: assessment of glycosylated hemoglobin levels and blood glucose values in arterial blood gas analysis. When abnormalities were detected, endocrinology consultation was requested for individualized treatment plans (oral hypoglycemic agents, subcutaneous insulin injection, insulin pump), with blood glucose monitoring before and after meals and at bedtime. (2) Postoperative blood glucose monitoring was performed immediately upon return to the ward. If blood glucose levels were elevated above normal, insulin pump therapy was initiated with blood glucose monitoring every 4 h.

TCM appropriate technology: Auricular seed pressure therapy combined with acupoint application therapy was implemented. (1) Auricular seed pressure therapy: Acupoints (stomach, large intestine, pancreas, endocrine, and other auricular points) were selected, and the ear skin was cleaned with ethanol. Vaccaria seeds were placed in the center of 0.5 × 0.5 cm medical adhesive tape and applied to the acupoints. Nurses applied appropriate pressure based on individual patient tolerance, generally until sensations of soreness, numbness, or pain were elicited. Pressure was applied for 3–5 min, 3–5 times daily. Both ears were treated simultaneously. The Vaccaria seeds and adhesive tape were replaced every 3 days or earlier if patients reported discomfort (e.g., skin redness or itching), and treatment continued for 14 consecutive days. (2) Acupoint application therapy: The hospital pharmacy provided an external herbal formula consisting of: *Artemisia argyi* (15 g), *Rheum officinale* (10 g), *Syzygium aromaticum* (5 g), Borneol (3 g), *Evodia rutaecarpa* (8 g), *Citrus aurantium* (10 g), *Magnolia officinalis* (10 g), *Bupleurum chinense* (10 g), *Ligusticum chuanxiong* (10 g), and *Cyperus rotundus* (10 g). The herbs were ground into powder and mixed with glycerin, yellow rice wine, and fresh ginger juice (in a ratio of 10:10:3) to form a paste. Thirty grams of the herbal powder was prepared as a paste and applied to the Shenque acupoint (CV8). After cleaning and drying the application site, the herbal paste was applied (covering an area of 10 × 10 cm with a thickness of 2 mm) and secured with medical film. Applications lasted 5 h per session, twice daily (9:00 and 16:00), for a treatment course of 14 days.

### 2.3. Blood Glucose Fluctuations

Blood glucose was monitored in both groups at different time points before and after surgery (1 day preoperatively, and postoperative days 1, 3, 7, and 14). Monitoring included preoperative seven‐point blood glucose measurements (fasting, premeal, and postmeal for three meals, and bedtime), postoperative monitoring every 4 h for Days 1–7, and a return to preoperative frequency after Day 7. Blood glucose fluctuation values were calculated for each time point, defined as the difference between the highest and lowest blood glucose values.

### 2.4. Comfort Assessment

The General Comfort Questionnaire (GCQ) [13], designed by Kolcaba in 1992, was used to assess comfort levels. This scale includes environmental, social, psychological, and physiological dimensions, with a total of 28 items. Each item uses a four‐point Likert scale (1–4 points), with reverse scoring for negative items and direct scoring for positive items. The total score ranges from 28 to 112 points, with lower scores indicating poorer comfort. The Cronbach’s *α* coefficient of this scale is 0.92, demonstrating high reliability. Assessment time points were 1 day preoperatively and 14 days postoperatively.

### 2.5. Stress Level Assessment

The Connor–Davidson Resilience Scale (CD‐RISC) [14] was used to evaluate psychological stress in both groups. This scale consists of three dimensions: tenacity (13 items), optimism (4 items), and self‐improvement (eight items), totaling 25 items. Each item is scored on a scale from 1 to 4. The total score ranges from 25 to 100, with higher scores representing stronger psychological resilience. For physiological stress indicators, serum adrenocorticotropic hormone (ACTH) and cortisol (Cor) levels were analyzed. Specific detection methods were as follows: 3 mL of peripheral venous blood was collected in the fasting state in the morning, serum was separated, ACTH levels were detected using an automatic biochemical analyzer, and Cor levels were detected by radioimmunoassay. Both physiological and psychological stress indicators were assessed 1 day preoperatively and 14 days postoperatively.

### 2.6. Perception of Nursing Service Quality

The Perception of Nursing Service Quality (PNSQ) scale was used to evaluate patients’ perceptions of nursing service quality in both groups. This scale is divided into four dimensions: medical environment (nine items), health education (nine items), service attitude (eight items), and nursing technique (three items), totaling 29 assessment indicators. Each dimension is scored on a scale from 0 to 4, with higher scores representing greater patient satisfaction with nursing services. Assessment time points were 1 day preoperatively and 14 days postoperatively.

### 2.7. Statistical Analysis

Experimental data were analyzed using SPSS 22.0 software. Normally distributed quantitative data were expressed as mean ± standard deviation ($\bar{x}\pm s$). Comparisons between groups were performed using independent samples *t* tests, while intragroup comparisons used paired *t* tests. Observations at different time points were compared using two‐factor repeated measures analysis of variance, with the LSD method for pairwise comparisons. Count data were expressed as rates (%), and intergroup comparisons used the *χ* ^2^ test. *p* < 0.05 was considered statistically significant.

## 3. Results

### 3.1. Comparison of Blood Glucose Fluctuation Values Between Groups at Different Time Points

Analysis of variance revealed significant between‐group differences, indicating that blood glucose fluctuation values differed significantly between the study group and control group (*p* < 0.05). Time point differences were also significant, demonstrating that blood glucose fluctuation values varied significantly across different time points (*p* < 0.05). The interaction effect between group and time point was significant (*p* < 0.05), suggesting that the differential trends in blood glucose changes between the study group and control group were not consistent over time, but were influenced by the interaction between the two groups. Specifically, the pattern of blood glucose changes in the study group at different postoperative time points differed from that in the control group. Further pairwise comparisons revealed that although blood glucose levels in both groups showed a downward trend, the study group exhibited significantly lower blood glucose levels than the control group at each time point (*p* < 0.05). See Table 1.

**Table 1:** Comparison of blood glucose fluctuation values between groups at different time points ($\bar{x}\pm s$).

**Group**	**n**	**Pre-op Day 1**	**Post-op Day 1**	**Post-op Day 3**	**Post-op Day 7**	**Post-op Day 14**
Study group	50	4.34 ± 0.65	5.15 ± 0.71^∗^	3.47 ± 0.46^∗^	3.05 ± 0.41^∗^	2.35 ± 0.42^∗^
Control group	50	4.54 ± 0.62	6.45 ± 0.68	4.21 ± 0.58	3.97 ± 0.53	3.54 ± 0.48
Between groups	—	*F* = 9.457; *p* < 0.001				
Time points	—	*F* = 6.542; *p* < 0.001				
Group.Time Points	—	*F* = 10.055; *p* < 0.001				

Note: Compared with the control group,  ^∗^ *p* < 0.05. Only the study group shows significant difference compared with the control group at each time point; no significant intergroup difference was observed for the control group′s data vs. the study group beyond the marked  ^∗^ items.

Abbreviations: Post‐op, postoperative; Pre‐op, preoperative.

### 3.2. Comparison of Postoperative Comfort Levels

On Day 1 before surgery, there were no significant differences between the two groups in the total GCQ score and scores in the four dimensions (*p* > 0.05). By Day 14 after surgery, the total GCQ score in the study group (89.36 ± 12.40) was significantly higher than that in the control group (73.07 ± 10.14), and the difference was statistically significant (*p* < 0.05). At Day 14 after surgery, the study group scored significantly higher than the control group in all four dimensions: environmental, social, psychological, and physiological (*p* < 0.05). Overall, comfort levels in both groups improved compared with preoperative levels, but the magnitude of improvement was greater in the study group. See Table 2.

**Table 2:** Comparison of postoperative GCQ scores between two groups (points, $\bar{x}\pm s$).

**Group**	**n**	**Environmental**		**Social**		**Psychological**		**Physical**		**Total score**	
		**Pre-op Day 1**	**Post-op Day 14**	**Pre-op Day 1**	**Post-op Day 14**	**Pre-op Day 1**	**Post-op Day 14**	**Pre-op Day 1**	**Post-op Day 14**	**Pre-op Day 1**	**Post-op Day 14**
Study	50	14.32 ± 1.98	20.15 ± 2.79^∗^	15.51 ± 2.14	21.32 ± 2.95^∗^	20.03 ± 2.77	33.14 ± 4.59^∗^	14.02 ± 1.94	14.75 ± 2.03	63.88 ± 8.85	89.36 ± 12.40^∗^
Control	50	14.25 ± 1.96	16.44 ± 2.28^∗^	15.33 ± 2.12	17.51 ± 2.42^∗^	19.94 ± 2.75	27.66 ± 3.83^∗^	14.17 ± 1.96	11.46 ± 1.59^∗^	63.69 ± 8.74	73.07 ± 10.14^∗^
*t*‐value		0.178	7.281	0.423	7.061	0.163	6.482	0.385	9.022	0.108	7.191
*p*‐value		0.859	< 0.001	0.674	< 0.001	0.871	< 0.001	0.701	< 0.001	0.914	< 0.001

Note: Compared with preintervention,  ^∗^ *p* < 0.05.

Abbreviations: GCQ, General Comfort Questionnaire; Post‐op, postoperative; Pre‐op, preoperative.

### 3.3. Comparison of Stress Response Levels

There were no significant differences between the two groups in CD‐RISC scores, ACTH, and Cor levels on Day 1 before surgery (*p* > 0.05). By Day 14 after surgery, the CD‐RISC scores in the study group were significantly higher than those in the control group, while ACTH and Cor levels were significantly lower than those in the control group. These differences were statistically significant (*p* < 0.05). See Table 3.

**Table 3:** Comparison of postoperative stress indicators between two groups ($\bar{x}\pm s$).

**Group**	**n**	**CD-RISC (points)**		**ACTH (ng/L)**		**Cor (ng/L)**	
		**CD-RISC (Pre-op Day 1, points)**	**CD-RISC (Post-op Day 14, points)**	**ACTH (Pre-op Day 1, ng/L)**	**ACTH (Post-op Day 14, ng/L)**	**Cor (Pre-op Day 1, ng/L)**	**Cor (Post-op Day 14, ng/L)**
Study	50	52.65 ± 7.31	74.23 ± 10.3^∗^	15.34 ± 2.12	12.23 ± 1.68^∗^	11.85 ± 1.64	9.46 ± 1.29^∗^
Control	50	51.95 ± 7.19	65.34 ± 9.06^∗^	15.46 ± 2.14	14.41 ± 1.98	11.69 ± 1.61	10.22 ± 1.41
*t*‐value		0.483	4.583	0.282	5.936	0.492	2.873
*p*‐value		0.630	<0.001	0.779	<0.001	0.624	0.005

Note: Compared with preintervention,  ^∗^ *p* < 0.05.

Abbreviations: ACTH, adrenocorticotropic hormone; CD‐RISC, Connor–Davidson Resilience Scale; Cor, cortisol; Post‐op, postoperative; Pre‐op, preoperative.

### 3.4. Perception Scores of Nursing Service Quality

There were no significant differences between the two groups in PNSQ dimension scores on Day 1 before surgery (*p* > 0.05). By Day 14 after surgery, the PNSQ dimension scores in the study group were significantly higher than those in the control group, and the differences were statistically significant (*p* < 0.05). See Table 4.

**Table 4:** Comparison of PNSQ scores between two groups (points, $\bar{x}\pm s$).

**Group**	**n**	**Medical environment**		**Health education**		**Service attitude**		**Nursing technique**	
		**Pre-op Day 1**	**Post-op Day 14**	**Pre-op Day 1**	**Post-op Day 14**	**Pre-op Day 1**	**Post-op Day 14**	**Pre-op Day 1**	**Post-op Day 14**
Study	50	24.57 ± 3.39	30.82 ± 4.26^∗^	24.15 ± 3.34	32.46 ± 4.49^∗^	22.53 ± 3.11	29.54 ± 4.08^∗^	7.35 ± 1.01	10.75 ± 1.48^∗^
Control	50	24.61 ± 3.41	27.75 ± 3.85^∗^	24.09 ± 3.18	28.57 ± 3.96^∗^	22.47 ± 2.95	26.94 ± 3.73^∗^	7.21 ± 0.99	8.86 ± 1.22^∗^
*t*‐value		0.059	3.781	0.092	4.595	0.099	3.326	0.700	6.968
*p*‐value		0.953	< 0.001	0.927	< 0.001	0.921	0.001	0.486	< 0.001

Note: Compared with preintervention,  ^∗^ *p* < 0.05.

Abbreviations: PNSQ, Patient Nursing Satisfaction Questionnaire; Post‐op, postoperative; Pre‐op, preoperative.

## 4. Discussion

The results of this study demonstrate that TCM appropriate technology shows multiple positive effects in the perioperative care of patients with esophageal cancer. The study group showed significantly better outcomes than the control group in terms of blood glucose control, postoperative comfort, stress response regulation, and perception of nursing services, providing important evidence for the application of TCM techniques in the perioperative period.

In terms of blood glucose control, the blood glucose fluctuation values at each postoperative time point in the study group were significantly lower than those in the control group (*p* < 0.05), which is consistent with the findings of Zhang et al., who also reported TCM’s role in improving postoperative glucose metabolism—specifically, that TCM interventions can enhance tissue glucose uptake and utilization to stabilize blood glucose levels [15]. Research has shown that TCM appropriate technology interventions (abdominal massage) can improve glucose metabolism in postoperative patients by regulating autonomic nervous system function. TCM appropriate techniques (abdominal massage, auricular stimulation) can effectively promote local blood circulation, increase tissue uptake and utilization of blood glucose, and enhance insulin secretion and action, thereby lowering blood glucose levels [16, 17]. Notably, there was a significant difference in the trend of blood glucose fluctuations between the two groups in this study (F = 10.055, *p* < 0.001). The study group showed a marked decrease in blood glucose fluctuations by the third postoperative day (3.47 ± 0.46 mmol/L), while the control group did not reach a similar level until the seventh postoperative day (3.97 ± 0.53 mmol/L). This difference is likely related to the regulatory effect of TCM appropriate technology on surgical stress response—supported by the study group’s significantly lower ACTH and Cor levels (physiological stress indicators) compared with the control group.

TCM appropriate technology reduces perioperative emergency responses, with ACTH and Cor levels in the study group significantly lower than those in the control group (*p* < 0.05), which is consistent with He et al.’s research on auricular acupressure reducing stress responses in surgical patients [18]. Surgical trauma activates the hypothalamic–pituitary–adrenal axis, leading to increased secretion of stress hormones, which in turn causes insulin resistance and elevated blood glucose [19]. TCM appropriate technologies (auricular seed pressure, herbal application) may regulate this neuroendocrine pathway, mitigating the adverse effects of surgical stress on glucose metabolism.

Regarding postoperative comfort, TCM appropriate technology helped improve patients’ postoperative comfort levels. The total GCQ score and scores in each dimension in the study group were significantly higher than those in the control group (*p* < 0.05). Particularly noteworthy was the improvement in the psychological dimension (study group 33.14 ± 4.59 vs. control group 27.66 ± 3.83), which was consistent with the trend in CD‐RISC scores. These findings suggest that TCM appropriate technology not only improved patients’ physiological condition but also significantly enhanced their psychological adaptability. TCM techniques (such as acupoint stimulation) regulate the hypothalamic–pituitary–adrenal axis, reducing cortisol (stress hormone) levels and decreasing the negative impact of chronic stress on psychological state [20]. TCM techniques activate the vagus nerve, enhance parasympathetic activity, and reduce excessive sympathetic excitation (such as palpitations, tension, etc.), thereby improving psychological adaptability [21]. Patients experience reduced fear and helplessness regarding their disease because of physiological changes such as improved blood glucose control and reduced pain, indirectly enhancing their confidence in coping with stress. Wu et al. also found that TCM therapy significantly improved the psychological state of surgical patients [22]. They selected thoracoscopic patients in thoracic surgery as research subjects, with similar intervention methods. They significantly improved the psychological state of surgical patients through TCM therapy (electroacupuncture), which corroborates our research results of improving patients’ psychological adaptability through TCM therapy (auricular acupressure combined with herbal hot compress). However, Wu et al.’s research focused on psychological scale assessment; whereas in our study, while focusing on psychological state, it also comprehensively evaluated multiple dimensions of patient comfort.

With the transformation of healthcare models, the impact of nursing service quality on patient rehabilitation outcomes and satisfaction has received increasing attention. Perception scores of nursing service quality, as an important indicator for evaluating the effectiveness of nursing work, are crucial for improving overall healthcare service levels. An important finding was the improvement in perception scores of nursing service quality. The improvement in the health education dimension was most significant (study group *Δ*8.31 points vs. control group *Δ*4.48 points), which may be related to enhanced nurse–patient communication during the implementation of TCM techniques. TCM appropriate technologies (such as acupoint massage, auricular acupressure) require deep interaction between healthcare providers and patients, with healthcare personnel needing to explain the operating principles, acupoint positioning, and precautions in detail before the procedure [23]. TCM health education often incorporates life‐like analogies (such as comparing “qi and blood blockage” to “pipe blockage”), making complex medical concepts easier to understand and improving patient comprehension [24]. Fawkes and Carnes [25] pointed out in their research that TCM techniques enhance service satisfaction. Their study also advocated using patient‐reported outcome measures to evaluate nursing effects, collecting not only the Bournemouth Questionnaire and Global Change scores but also data on patient satisfaction and experience with care. The study showed that patients receiving TCM treatment had high scores for satisfaction and experience with care.

The limitations of this study include the following: First, the sample size is relatively small and the study was conducted at a single medical institution, so the generalizability and reliability of the results need further verification. Future research could consider expanding the sample size and conducting multicenter studies to more comprehensively evaluate the effects of TCM nursing interventions. Second, while this study has preliminarily explored the specific content and operation methods of TCM nursing interventions, it has not conducted an in‐depth analysis of the independent effects of different intervention measures, specifically different TCM appropriate technologies (such as auricular seed pressure, acupoint application therapy). Future research could further explore the effectiveness and applicability of various intervention measures to develop more scientifically sound TCM nursing plans. Additionally, this study primarily focused on the effects during the postoperative recovery period and has not observed long‐term follow‐up results. Future research could extend the follow‐up time to observe the impact of TCM nursing interventions on patients’ long‐term quality of life and functional status, thereby more comprehensively evaluating their clinical value. Finally, this study did not evaluate the cost‐effectiveness of TCM nursing interventions, which is important for policy‐making and guiding clinical practice. Future research could incorporate cost‐effectiveness analysis to comprehensively evaluate the economic and social benefits of TCM nursing interventions, providing stronger support for their promotion and application in clinical practice.

## Ethics Statement

The study protocol was approved by the Ethics Committee of Nanjing Medical University and was conducted in accordance with the Declaration of Helsinki. Written informed consent was obtained from all patients or their legal representatives prior to enrollment in the study. Patients were fully informed about the purpose, methods, potential benefits, and risks of the study. Participation was voluntary, and patients were informed of their right to withdraw from the study at any time without affecting their standard care. All patient data were anonymized and handled confidentially to protect privacy.

## Conflicts of Interest

The authors declare no conflicts of interest.

## Author Contributions

Ying Fang conceived and designed the study. Jifang Peng, Dandan Yang, and Hairong Liu collected and analyzed the data. Tianle Chen performed the statistical analysis. Juan Cao supervised the project. Ying Fang drafted the manuscript. All authors contributed to the interpretation of the results, critically revised the manuscript for important intellectual content, and approved the final version of the manuscript.

## Funding

No funding was received for this manuscript.

## Data Availability

The data that support the findings of this study are available from the corresponding author upon reasonable request.
